# Attitudes of pediatric intensive care unit physicians towards the use of cognitive aids: a qualitative study

**DOI:** 10.1186/s12911-016-0291-6

**Published:** 2016-05-21

**Authors:** Matthew J. Weiss, Chelsea Kramer, Sébastien Tremblay, Luc Côté

**Affiliations:** Division of Pediatric Critical Care, Centre Mère-Enfant Soleil du Centre Hospitalier Universitaire de Québec, 2705 boul Laurier Local R1735, Québec, QC G1V 4G2 Canada; Department of Pediatrics, Université Laval, Faculty of Medicine, Québec, Canada; School of Psychology, Université Laval, Faculty of Social Sciences, Pavillon Félix-Antoine-Savard, 2325, rue des Bibliothèques, Québec, G1V 0A6 Canada; Department of Family and Emergency Medicine, Université Laval, Faculty of Medicine, Pavillon Ferdinand-Vandry, 1050, avenue de la Médecine Local 2207A, Québec, G1V 0A6 Canada

**Keywords:** Cognitive aids, Physician attitudes, Qualitative research, Pediatric intensive care, Theory of planned behavior, Implementation

## Abstract

**Background:**

Cognitive aids are increasingly recommended in clinical practice, yet little is known about the attitudes of physicians towards these tools.

**Methods:**

We employed a qualitative, descriptive design to explore physician attitudes towards cognitive aids in pediatric intensive care units (PICUs). Semi-structured interviews elicited the opinions of a convenience sample of practicing PICU physicians towards the use of cognitive aids. We analyzed interview data for thematic content to examine the three factors of intention to use cognitive aids as defined by the Theory of Planned Behavior (TPB), attitudes, social norms, and perceived control.

**Results:**

Analysis of 14 interviews suggested that in the PICU setting, cognitive aids are widely used. Discovered themes related to their use touched on all three TPB factors of intention and included: aids are perceived to improve team communication; aids may improve patient safety; aids may hinder clinician judgment; physicians may resist implementation if it occurs prior to demonstration of benefit; effective adoption requires cognitive aids to be integrated into local workplace culture; and implementation should take physician concerns into account.

**Conclusions:**

Our sample of PICU physicians were open to cognitive aids in their practice, as long as such aids preserve the primacy of clinical judgment, focus on team communication, demonstrate effectiveness through preliminary testing, and are designed and implemented with the local culture and work environment in mind. Future knowledge translation efforts to implement cognitive aids would benefit from consideration of these issues.

## Background

The term cognitive aid includes a wide range of tools designed to reduce mental burden on memory and complex decision making. These tools have been variously defined as “techniques and principles that have emerged to help people to detect, interpret, store and retrieve information efficiently” [1] or more recently and specifically for the medical field, “structured pieces of information designed to enhance cognition and adherence to medical best practices” [2]. They are available in many forms and settings ranging from a simple household recipe to the various checklists and prompts airline pilots use during emergency landings [3]. The potential benefit of cognitive aids for physicians has been the subject of increasing interest [4–9]. Demonstrated benefits seen with medical cognitive aids include reduced surgical mortality with the use of a pre-operative checklist [6], contribution to marked reduction in central line infection rates in adult intensive care units [4], improved information transfer at handover of Pediatric Intensive Care Unit (PICU) patients [7, 8], and decreased omission errors in preparation of emergency pediatric intubation [10]. A 2009 review of a specific type of cognitive aids in the PICU, computerized decision support systems (CDSS), found them to be a promising tool, but one that required further study [11]. This growing body of experimental evidence suggests a role for cognitive aids, but, as discussed below, there is limited published research about physician attitudes towards these tools.

Research on the successful implementation of cognitive aids has stressed that simple development and delivery of cognitive aids is often inadequate. In a landmark study on central line insertion bundles that included cognitive aids from Berenholtz et al., researchers reported the positive effect of having senior hospital administration officials implicated early in the implementation and oversight of the cognitive aid project [4]. In a later editorial, including a senior author of that study, the importance of the “simple” checklist cognitive aid itself was deemphasized relative to culture changes and the need for local adaptation [12]. Similarly, while the Surgical Safety Checklist developed by the World Health Organization (WHO) resulted in impressive improvements in surgery mortality, the team that initially implemented this cognitive aid made extensive site visits to participating centers to ensure proper usage and adequate uptake of the aid [6]. Implementation of the same WHO surgical checklist has shown variable effectiveness outside of the initial project, as documented in Guatemala City [13] and across the UK [14]. These examples suggest that understanding of local factors and attitudes towards cognitive aid use might play an important role in their effectiveness.

To date, the attitudes of physicians towards cognitive aids, and how these attitudes might affect adoption of cognitive aids have not been extensively reported. Qualitative studies of attitudes towards the WHO checklist, have revealed both positive and negative reactions among health care providers. These include improved standardization of practice in unfamiliar settings [15], but also strong resistance from senior physicians [8], and concerns over workflow interruption [14, 15]. A recent online survey of anesthesia providers found that most were supportive of some forms of cognitive aids, though distraction and efficiency concerns were frequently cited concerns, especially in regards to cognitive aid use in routine settings [16]. These data are informative but limited, and there is a gap between knowledge related to the effectiveness of cognitive aids and physician attitudes towards their usage and implementation. This gap potentially limits knowledge translation strategies for cognitive aid adoption.

The objective of our study is to address this knowledge gap by examining the attitudes of physicians in a specific clinical setting, the PICU. The PICU is an environment that involves high patient turnover, patients from widely variable age groups (from a few days to 18-years-old in most centers), and management of diverse, life threatening pathologies. Clinicians in this setting must manage a large volume of physiologic data while dealing with the logistic and emotional stresses of caring for very ill, unstable children. This environment is precisely the type of high risk, high distraction environment in which cognitive aids have been demonstrated to be effective [17]. While cognitive aids have been developed and shown to be beneficial in this setting, the above reports suggest that without study of physician attitudes towards them, barriers to implementation will remain [11].

In order to better understand physician attitudes towards cognitive aids in this setting, we applied qualitative methodology based in the Theory of Planned Behavior (TPB) as our theoretical framework [18]. As described in a review by Godin et al., the TPB has been widely used to investigate attitudes and how they influence the adoption of behaviors and been shown to robustly predict future behavioral change among health care professionals [19]. Since not all the cognitive aids we were analyzing were technologically based (e.g. paper algorithm cards were included) we chose not to apply other models such as the technology acceptance model [20]. According to the TPB, intention to adopt or perform a behavior (e.g. cognitive aid use) is a major determinant, and requisite first step of behavior adoption. Intention is determined by a) the person’s *attitude*, favorable or unfavorable, towards adopting the behavior, b) the *social norms*, or perception of the social pressure to adopt the behavior, and c) the *perceived behavioral control*, or their perception of how easy it might be to adopt the behavior [18]. In order to inform future design choices regarding cognitive aids in the PICU we also queried participants as to what futures they most desired in their cognitive aids.

## Methods

### Study design

We employed a descriptive, qualitative research design with thematic content analysis of semi-structured interviews of practicing PICU physicians.

### Participants and recruitment strategy

The first author (MW) recruited 15 participants from his professional network of PICU physicians working across Canada. Physicians were invited to participate via direct email or through their division director. This email included an information and consent form, with verbal consent obtained at the time of the interview. Our purposeful sampling approach sought participant diversity according to age, gender, primary language, experience, and practice locations. At least one physician from each of the six contacted centers agreed to participate. Interviews were anonymized and the audio recordings and transcriptions were stored on a password protected computer. This study was approved by the research ethics board of the Centre Hospitalier Universitaire de Québec.

### Data collection

MW conducted individual semi-structured phone interviews in either French or English, depending on the preference of the participant. The interview grid included nine main questions (see Appendix 1), accompanied by follow-up or clarification questions. As described by Francis et al. [21], the interview was based on the TPB as a theoretical framework with questions addressing attitudes towards the use of cognitive aids, social norms around cognitive aid use, and their perception of control over their capacity to adopt cognitive aid use themselves or influence their use by others. At the end of the interview, and separate from the TBP framework, we asked participants what features they would desire from cognitive aids designed to assist with their practice. Two investigators (MW and LC) developed the grid using clinical experience in a PICU environment (MW) and expertise applying the TPB for qualitative medical research (LC) [22, 23]. To ensure that the interview was comprehensible and well organized, the first two interviews served as pilot interviews. For this process, participants were asked to provide feedback on the clarity and structure of the questions. Interviews were also analyzed for interviewer interaction with participants. Following these pilot sessions, minor revisions were made to the grid including changes to question order, and a reduction in perceived repetition of interview themes. Since pilot participants expressed good understanding of the research goal and questions, and MW and LC judged that their responses did not substantially differ from later participants, their responses were included in the final analysis. All interviews were audio recorded and transcribed verbatim by trained research assistants.

### Data analysis

Interview transcriptions were coded independently by a research assistant and two researchers (MW and LC). An inductive analysis was applied, with new codes created each time a participant was noted to have given a novel response to an interview questions. If a concept had already been mentioned by another participant, that statement was assigned a pre-existing code. The interviews were analyzed by the coders individually, and then in meetings where the three coders compared their results. One to three interviews were coded at each meeting. In cases of disagreement, all three returned to the transcripts and lists of codes in order to generate consensus. During analysis MW provided content expertise and explanation of clinical scenarios, where LC provided expertise in qualitative analytic methods. This iterative process led to the creation of new codes, improvement of code definitions, and code merging. Any merging of codes was also done by consensus among MW and LC when judged to contain sufficient overlap. Data saturation was monitored by evaluating the frequency of new codes with subsequent interviews. Once saturation was achieved, the frequency that each code was mentioned was noted. LC and MW then grouped codes into themes based on code title and the messages conveyed by participant quotations. Considering the qualitative nature and small sample size, no demographic-based comparisons were planned or performed, though basic participant demographic data was collected.

## Results

Fifteen physicians agreed to participate and were interviewed. As some invitations were sent to directors of PICU divisions, it is unclear how many physicians were contacted, thus a response rate is impossible to calculate. One interview was lost through corruption of the electronic audio file. Demographic data of the remaining 14 participants are provided in Table 1. Interviews lasted from 18–47 min (average 31.4 min.).

**Table 1:** Participant Demographics

Gender	
Male	9
Female	5
Years of Staff Experience	
0–5	3
5–10	6
10–15	1
> 15	4
Working Language	
English	5
French	4
Mixed English/French	4
Unit Size (Number of beds)	
0–10	4
10–15	3
> 15	7
Type of Unit	
Medical/General Surgery	3
Cardiac Surgery	1
Mixed	10

Thematic saturation was achieved with few to no novel concepts discovered in analysis of the final three interviews. We grouped the identified themes according to the relevant TPB factors of attitude, social norms and perceived control. Table 2 lists the eight major themes discovered using this analysis. Since some of the themes addressed more than one TPB factor of intention, these themes are discussed more than once in their respective TPB factor sections.

**Table 2:** Discovered Themes Sorted by TPB Factor

TPB Factor	Major Themes
Attitudes	Cognitive aid use might limit physician judgment
Attitudes	Cognitive aid use might improve patient safety
Attitudes/Social Norms	Cognitive aids are widely used in a PICU setting
Attitudes/Social Norms	Cognitive aids are seen to improve team communication and crisis resource management skills
Attitudes/Social Norms	Need to demonstrate cognitive aid efficacy
Social Norms	Family presence at bedside would not influence cognitive aid use
Social Norms/Perceived Control	A culture of safety incorporating cognitive aid use is important for their adoption and continued use
Perceived Control	Requirement of cognitive aid use by administration or regulatory bodies has positive and negative effects

### Attitudes towards cognitive aids

As one of the major determinants of intention in the TPB framework, attitudes towards an intervention are key prior to acceptance of a new behavior. Several of the questions in the questionnaire were targeted at eliciting participant attitudes toward the use of cognitive aids in their PICU practice.

### Widely used in a PICU setting

All participants reported using some form of cognitive aid in their personal lives, and 10/14 respondents reported using some form of cognitive aid in their professional lives. The types of professional aids mentioned by participants are summarized in Table 3. Cognitive aids were frequently cited as useful in situations that are rare, error prone, high risk or stressful. For example, 13/14 participants perceived cognitive aids such as PALS cards as useful in resuscitation settings. One participant mentioned that “when people are stressed and they’re not thinking things clearly, there are a lot of things that I know right now that I can tell you and then, if I’m stressed out, I’m going to start forgetting little details.”

**Table 3:** Cognitive Aids Actively Used By Participants In PICU Practice

Care bundles^a^	
Pals algorithm cards	
Personal checklists for rounds	
Printed standardized order sets^b^	
Smart phone medical applications	
Handover or discharge checklists	
National or local clinical practice guidelines	
Preprinted resuscitation medication calculation sheets	
Reminders embedded in electronic medical records or computer order entry systems	

^a^– Kits that include checklists and necessary materials to perform procedures or clinical tasks (e.g. central line bundles)

^b^– Order sets for specific clinical scenarios (e.g. admission of a diabetic patient or start of hemodialysis)

Aids were frequently mentioned as useful for clinicians with little experience in a PICU setting (7/14). Other situations where aids were judged useful included (one respondent each): repetitive clinical settings, PICUs where clinical practice varies significantly between practitioners, clinical settings where the evidence base is well developed, and environments with a high risk of distraction.

### Improved care team communication – standardization of practice

The majority (10/14) of participants mentioned the benefits of standardization of practice within a team, with a subsequent decrease in communication confusion between physician and allied health team members. One participant stated, “I think that for uniformity of clinical practice, I find that desirable…Especially in a unit where doctors change service at night and on weekends. If these doctors have completely different practices…that can lead to confusion on the part of the nursing staff.” Thus, cognitive aids were seen by some as tools that encourage uniform, accepted practices throughout a unit.

### May improve patient safety

A majority (10/14) of participants also mentioned that aids can decrease the amount of forgotten details, and 6/14 thought this could lead directly to improvement in patient safety. Some (5/14) cited complex bedside rounds with multiple, routine details that are easily forgotten, but that could be collated into a daily checklist. These potential advantages were to thought to be particularly useful for junior physicians or learners by 7/14 respondents. One senior participant summarized the attitude that while the patient safety benefits might be present for all physicians, less experienced users might benefit the most by stating: “It’s not something that I use commonly. Though, I expect people junior to me to use them, which I kind of find entertaining now that I say it out loud.” Creation of these aids was also seen by one participant as a convenient way to summarize complex literature and make it available for routine decision making.

### May limit clinical judgment

The most frequently perceived disadvantage to PICU cognitive aid use was limitations to physician reflection or judgment (7/14). There was a sense that aids could become a distractor from the patient’s actual clinical condition (3/14), or impede reaction time (2/14). Participants mentioned that aids might create a dependence on the tool, inhibit learning or decrease effective memorization. One participant said that that “the perverse effect of all these aids is that you prohibit your brain to reflect because everything becomes a little automatic.” Two participants mentioned that these limitations might pose a risk to patient safety if the aid limited judgment to the point of applying an inappropriate protocol. Concern over loss of clinical judgment was also discussed in terms of the design of future cognitive aids. Some participants objected to the requirement of following a protocol (6/14). One participant said, “I would say I’m for it, with the limit that you always need to leave room for clinical judgment because these things are always going to be susceptible to technical errors.”

### Need to demonstrate cognitive aid efficacy

Participants expressed a desire to examine evidence supporting cognitive aid use in multiple ways. Several stated that if there was a clear scientific consensus that use of cognitive aids improved patient outcomes, they should be adopted, and possibly even imposed by regulators or hospital administers (8/14). Even those supporting that position, however, cautioned that such evidence should include outcomes on all aspects of cognitive aid use, such as the respondent who stated that she would like to see “what is the impact on the entire PICU system, including nursing and physician time.” Similarly, some participants stated they would be opposed to cognitive aid use without data supporting their efficacy (3/14)

#### Social norms related to colleague cognitive aid use

Social norms are the second major determinant of intention described in the TPB. The perception of how a behavior is perceived by one’s peers is an important consideration toward whether or not to adopt that behavior. The following responses were elicited by questions designed to examine social norms related to cognitive aid use in a PICU.

### Widely used in a PICU setting

Most (11/14) respondents reported frequent cognitive aid use by themselves and their colleagues. The most common formats included standardized orders, or checklists for techniques such as extracorporeal membrane oxygenation (a high risk therapy of mechanical cardiopulmonary bypass employed for extremely ill patients). Most expressed no opinion towards this use by their colleagues, though 2/14 had favorable opinions such as the participant who said, “I don’t think less of anybody who looks at a cognitive aid, in fact, I think more of them. I think that it’s just admitting. ‘Yeah I’m human, I can’t remember, I’ve got to look it up’.”

### Culture of safety incorporating cognitive aids – both encouraged and discouraged by colleagues

Most (8/14) participants had at least one colleague who had expressed positive opinions towards the use of cognitive aids by encouraging their use or pointing out their benefits. Several described having cognitive aid “champions” in their units, who often encouraged cognitive aid usage as a way to improve patient safety (4/14).

Several, however, had heard negative opinions towards cognitive aids efficacy expressed by their colleagues, specifically skepticism towards cognitive aids’ effectiveness (5/14). One participant even reported, “I think that sometimes it can even be a subject of a little bit of teasing, because there are some for who using a cognitive aid really isn’t their style.”

### Family presence would not influence cognitive aid use

Participants almost uniformly reported that the presence of a family at the bedside would not influence cognitive aid use (12/14). Half of those who would not be influenced by the family stated that it would increase transparency towards the family, including a participant who said, “I think it sends a strong message of attention to details and focus on patient safety to the family.” Still, a few participants expressed concerns that families might perceive them as incompetent if they were obligated, in the words of one participant, to “pull out my little checklist” in the treatment of a critically ill child (3/14).

### Might improve team communication – reduction of team hierarchy

Nearly all physicians said they would support an aid that would encourage non-physicians to remind physicians of discovered risks to patient safety (13/14). This finding was in line with other comments, such as a participant who mentioned that the presence of cognitive aids seems to diminish the effect of the professional hierarchy, allowing allied health personnel to more freely question physician treatment plans. This possible reduction of hierarchy extended to resuscitation teams, where non-physician team members using an aid might feel more comfortable to point out deviations in treatment algorithms (3/14). Nearly all who saw this as advantageous agreed with the participant who stated, “it doesn’t matter who recognizes a problem, if there’s a problem, it needs to be addressed.”

#### Perceived control

Several questions focused on how participants had responded to the implementation of cognitive aids in their work environments. Consistent with the TPB, success or failure of these strategies depended less on the specific methods used, and more on fostering a sense of ownership or perceived control over the development and implementation of a proposed cognitive aid.

### Culture of safety incorporating cognitive aids

Several participants noted that if a cognitive aid initiative was developed by an individual in their group, and adopted into the culture of their unit, it was more likely to be successful (6/14). One such champion stated, “I think of all the enthusiasm, maybe that’s a little bit catchy but I’m really enthusiastic about it so eventually, I think maybe they’ll just jump on the train.” Others stated that progressive change with gradual implementation was important for aids to be integrated into the culture of a unit (5/14), with one stating that carefully integrated, well designed tools have become indispensable for several members of her colleagues. Two participants placed particular emphasis on the culture of a unit around cognitive aids including one who stated, “the cognitive aid in itself is just a piece of paper, but if it doesn’t go with the whole culture of the patient safety in the process of using it, I imagine nothing will change.”

When asked specifically about their perceived control over influence on their colleagues, nearly all felt that they had or could influence their colleagues around cognitive aid use (12/14), and only one specifically stated he or she could not. Methods used to influence others included demonstrating evidence that cognitive aids improve clinical performance or outcomes (4/14), acting as a role model (3/14), and generally promoting patient safety (2/14).

### Requirement of use by administration or regulatory bodies

Participants were generally less receptive to methods that involved imposition of tools by hospital administrators or regulatory bodies. For the interview, imposition was defined as requirements from a body outside of the physician group with the authority to require cognitive aid usage. Expressed concerns included that these tools would limit practitioner autonomy (3/14), be implemented without physician consultation (3/14), or not respect the realities of clinical workflow (2/14). One respondent discussing frustration with imposed tools that did not fit his needs in a quaternary medical center stated, “none of us practice in the median. I don’t want to be forced to practice in the median.”

Many participants, however, were open to the idea that administrators and regulators should have the authority to impose tools when compelling evidence demonstrates that cognitive aids improve adherence to proven best clinical practice (8/14). In the case of a hospital or enterprise level cognitive aid, two stated that imposition by administrators was an effective method to standardize practice, and two others pointed out that this method was one of few that would force physician adherence. One participant noted that without evident consequences for non-adherence, uptake by those resistant to aids would be slow. Further, implementation without evidence supporting their use was a significant barrier for participants (3/14). One participant mentioned worry over possible medico-legal risk if a physician deviated from cognitive aid recommendations, especially if that aid been imposed by a hospital or regulatory authority.

#### PICU physician expectations regarding cognitive aid design

As mentioned above, participants were also asked what they would ideally desire in future PICU cognitive aids. Most stated that electronic aides with incorporated real-time physiological data would likely represent an improvement over existing cognitive aids (e.g. PALS cards) (11/14). They also hoped that future aids would be able to link clinical situations to appropriate algorithms and relevant data from patient charts (4/14). That information would preferably be updated in a continuous manner (3/14), and possibly used to automatically predict imminent changes in patient status (2/14) or detect treatment omissions (2/14). Recommendations made by such a tool should be clear and directive (2/14).

Participants also hoped that aids would be specifically designed to limit distractions or fixation errors, since any tool intended for a resuscitation would have be used by someone in a high stress state (3/14). Any design should consider the risks of information overload (5/14) or inflexible tools (3/14), and avoid overly complex designs (2/14). Other significant design concerns were around issues of technical or software errors (5/14), and increased alarm fatigue (3/14).

## Discussion

As data accumulates suggesting the utility of various forms of cognitive aids in clinical practice, it is increasingly important to explore physician attitudes in order to design knowledge translation strategies for their adoption in practice. This study represents one of few such published explorations, and the first to our knowledge in the PICU. Using TPB grounded qualitative analysis, eight major themes emerged reflecting positive and negative attitudes, social norms and perceived control related to cognitive aid use. Overall, participants supported the use of cognitive aids in a wide variety of clinical settings, though with several expressed concerns.

The first major theme was the widespread use various forms of cognitive aids by PICU physicians. In the context of the TPB, this suggests that participant attitudes and social norms towards these tools are positive towards their incorporation. However, not all participants reported that colleagues encouraged cognitive aid use and some even reported active discouragement such as mild teasing of users. In their survey of cognitive aid use by anesthesia practitioners, Kromback et al. also reported that respondents, particularly junior providers, often felt less inclined to use cognitive aids in front of colleagues [16]. Concerns about implementation resistance due to entrenched cultural resistance led the team at Stanford University to directly engage the anesthesia culture when implementing their cognitive aid manuals for perioperative care [24]. Our findings are consistent with these studies and demonstrate that addressing the underlying safety culture to include cognitive aids will be important for future implementation efforts.

This is closely related to the second major theme which was that these tools are perceived to help with team communication. While communication was perceived to improve in a variety of settings, two particularities merit further discussion. The frequent use of cognitive aids seemed to decrease the perception of a professional hierarchy. Previous reports show that nursing staff felt more at ease addressing safety concerns when using a cognitive aid [15], which is in line with findings from our study. The second major communication issue was the potential of cognitive aids to improve crisis management communication. Crisis situations arrive frequently in a PICU environment, and participants noted potential advantages to aid use such as decreasing team fixation errors and improving adherence to established protocols. While this advantage to cognitive aid use has not been directly demonstrated, teams are developing methods to incorporate cognitive aids with the direct goal of improving team communication, not simply fact based recall [25].

While the above themes suggest support for increasing cognitive aid use, the was also a fear that these tools might limit clinician judgment. Many participants expressed concerns that a poorly adaptive or designed cognitive aid might push users to apply standard solutions to non-standard situations. This concern is supported by findings in at least one study of simulated pediatric resuscitation where, despite high rates of cognitive aid use, 25 % of residents using a cognitive aid chose the wrong treatment algorithm resulting in inappropriate management [26]. Attempts to generate useful PICU cognitive aids must keep in mind the importance of preserving clinician judgment and autonomy. Studies on attitudes towards implementation of a CDSS in Malaysia [27], Finland [28], and Ireland [29] found that threat to physician autonomy was a factor strongly associated with resistance to uptake. Training and implementation could also be developed to educate teams on how to understand the strengths and limitations of cognitive aid use. Recent reports on anesthesia cognitive aids also support increased training, suggesting even that cognitive aid use should be incorporated as a core residency competency [16, 24, 30]. In the TPB framework, this approach would likely create early attitude changes and increase social acceptance of these tools.

Another major attitude that limits the uptake of cognitive aid is the perceived lack of proof of efficacy. Participants stressed that, as with any practice change, expending the resources necessary to incorporate cognitive aids into practice should only be done if they are proven effective. This included skepticism expressed by other, often senior colleagues over their use, and personal reluctance to use a time consuming tool. These findings are consistent with reluctance from senior staff towards surgical checklists reported in interviews [14]. Testing in simulation prior to clinical use, as well as post introduction follow up to ensure that the tool performs as predicted would likely help address some of these concerns.

The final major themes in our study suggest any administration or regulating body must carefully consider physician attitudes and workplace culture prior to implementing of a cognitive aid. Similar to findings reported by Russ et al. [14], our participants reported resistance to imposition without an implementation plan designed to ease incorporation of an aid into the daily work flow. Conversely, participants expressed that having a governing body mandate use of an aid is often the only way to ensure uptake by physicians. These ideas were consistent with those published by other groups [4, 6, 12] who have emphasized that participation of senior administrators is key for long term successful implementation of a cognitive aid program. Our participants also strongly and repeatedly suggested that any hospital or regulatory body planning to implement a cognitive aid should foster a local patient safety culture encourages cognitive aid use. These findings align with the TPB concepts of social norms and perceived control, with uptake more likely when the use of a cognitive aid is seen as a required social norm, but where physicians retain a sense of control over adapting the aid to their particular work environment.

Participants consistently expressed desire for cognitive aids that could integrate existing patient information from monitors and electronic records with suggestions from protocols and algorithms while expressing concerns about information overload or useless reminders. These considerations stress the well documented though rarely heeded importance of incorporation of cognitive design principles into the development of clinical cognitive aids [30]. This would require careful testing of tools to ensure that they limit fixation errors, avoid alarm fatigue (a well-documented problem, even in pediatrics [31]), and present information in a way that preserves physician judgment.

### Limitations

The nature of our study design did not include observations, so actual use of cognitive aids could have been over or underestimated. Though the sample size was small, it represented a diverse group of Canadian PICU practitioners with a wide range of experience and types of units in which they practice. Results were monitored for saturation with each the final interviews yielding few or no new themes. The interview grid was a novel tool and underwent limited pilot testing. Pilot participants, however, were explicitly probed regarding comprehension of questions and face validity of the project, and reported no significant concerns. No control was made for the fact that the initial interviews had a slightly different order of questions, though their responses were judged to be substantially similar to those of other participants. The interviews were all conducted by MW in either French or English depending on the choice of the participant. While practicing full time in a francophone environment, his first language is English and it is possible that some subtleties might not have been fully explored during the interview. To compensate, all interviews, were analyzed by at least one native French speaker. Finally, while cognitive aid use in a PICU often effects all members of a multi-disciplinary team, the scope of this study was limited to the physician members of that team.

Despite these limitations, this study represents the first known exploration of attitudes towards cognitive aid use in a PICU environment. Considering the high stakes, highly interruptive environment of a PICU, this setting is an ideal candidate for aids that could diminish its cognitive burden. This benefit, however, is likely to limited to if cognitive aids are not designed that respect the factors that will lead to physician uptake. Well designed studies are needed to demonstrate the advantages of any newly proposed cognitive aid, both according to medical and implementation factors. Our findings also suggest that implementation would likely benefit from an iterative process, where feedback from the end users is incorporated in a way that allows for modification within the local culture and workflow.

## Conclusions

Our sample of PICU physicians were open to well-designed cognitive aids in their practice. This openness is contingent on the ability of such aids to preserve the primacy of clinical judgment, focus on team communication, have preliminary testing demonstrating their effectiveness, and be designed and implemented with the local culture and work environment in mind.

### Ethics and consent

This study was approved by the research ethics board of the Centre Hospitalier Universitaire de Québec. All participants gave verbal consent prior to participation. No identifying information related to participants was published, and the original recordings of the interviews will be destroyed 1 year after publication.

### Consent to publish

All participants gave consent to publish anonymized quotes as part of the final manuscript. Participants did not review the final manuscript, but were made aware of publication.

### Availability of data and materials

Considering that the transcripts and analysis of the research interviews contain data that could possibly identify participants, and that publication of the original data set was not included either in the ethics submission nor in the participant consent, this data has not been included in a public database. If further information regarding the data set is desired, please contact the corresponding author, who will discuss approval from his ethics board and participants for data sharing.

## Appendix

### Appendix 1: Interview Form

#### Introduction:

‘Cognitive aids’ is a term that covers a wide range of tools used in various settings. In the clinical realm it could include anything from a checklist used prior to surgery to the PALS resuscitation cards given out to residents. A growing body of literature is examining their use in various clinical settings, but few studies have explored the attitudes of physicians towards these tools. The goal today is simply to ask some questions about your views related to cognitive aids. Any questions before we get started?

1. (A) Do you use any form of cognitive aids in your everyday practice?
  - If yes: What cognitive aids do you use in your everyday practice?
  - If yes: What settings do you use the checklists in?

1. (A) What do you think about cognitive aids in clinical practice?
  - If not explicitly addressed in answer ask: How did you come to feel this way?
  - Is there a situation where cognitive aids would be particularly helpful? intrusive?
  - How do you feel about cognitive aid usage in acute settings? What might limit their usage in these settings? Conversely, what might facilitate their use?

1. (SN) Does your administration, hospital, department or division, encourage or discourage the use of cognitive aids?
  - If yes: What strategies have they employed? What did you think about these strategies?
  - Do you have any thoughts on whether an administration should or shouldn't require cognitive aid use?

1. (SN) Do any of your colleagues use cognitive aids? If yes what type of ICU professional? How regularly in what context?
  - How do you feel towards the fact that your colleagues do or don't use cognitive aids?
  - Do your colleagues approve/disapprove of the use of cognitive aids?

1. (SN) Would the presence of patient family members alter your tendancy to use or not use a cognitive aids?

1. (PC) Have you ever been part of a team where the use of a cognitive aid in a clinical setting was required by your administration or director?
  - How did that mandate make you feel?
  - What made you accept or reject the mandate?

1. (A) Do you think the use of cognitive aids might impact the team work of your ICU?

1. (PC) Do you feel like you have control over whether or not you use of cognitive aids?
  - What factors decrease or increase your sense of control over the use of these aids?
  - Do you feel you have the power to change whether or not cognitive aids are used in your environment?

1. Do you have anything to add or final comments?

## Abbreviations

CDSS: computerized decision support systems
PALS: pediatric advanced life support
PICU: pediatric intensive care unit
TPB: theory of planned behavior

## References

[1] Rosenthal TL, Downs A (1985). Cognitive aids in teaching and treating. Adv Behav Res Ther.

[2] Stanford School of Medicine - Cognitive Aids in Medicine [http://cogaids.stanford.edu/whatarecogaids.html] Accessed 11 May 2016.

[3] Gawande A (2010). The Checklist Manifesto.

[4] Berenholtz SM, Pronovost PJ, Lipsett PA, Hobson D, Earsing K, Farley JE, Milanovich S, Garrett-Mayer E, Winters BD, Rubin HR, Dorman T, Perl TM (2004). Eliminating catheter-related bloodstream infections in the intensive care unit. Crit Care Med.

[5] Garg AX, Adhikari NKJ, McDonald H, Rosas-Arellano MP, Devereaux PJ, Beyene J, Sam J, Haynes RB (2005). Effects of computerized clinical decision support systems on practitioner performance and patient outcomes: a systematic review. JAMA.

[6] Haynes AB, Weiser TG, Berry WR, Lipsitz SR, Breizat A-HS, Dellinger EP, Herbosa T, Joseph S, Kibatala PL, Lapitan MCM, Merry AF, Moorthy K, Reznick RK, Taylor B, Gawande AA, Safe Surgery Saves Lives Study Group (2009). A surgical safety checklist to reduce morbidity and mortality in a global population. N Engl J Med.

[7] Zavalkoff SR, Razack SI, Lavoie J, Dancea AB (2011). Handover after pediatric heart surgery: a simple tool improves information exchange. Pediatr Crit Care Med.

[8] Weiss MJ, Bhanji F, Fontela PS, Razack SI (2013). A preliminary study of the impact of a handover cognitive aid on clinical reasoning and information transfer. Med Educ.

[9] Hagerman NS, Varughese AM, Kurth CD (2014). Quality and safety in pediatric anesthesia. Curr Opin Anaesthesiol.

[10] Long E, Fitzpatrick P, Cincotta DR, Grindlay J, Barrett MJ: A randomised controlled trial of cognitive aids for emergency airway equipment preparation in a paediatric emergency department. Scand J Trauma Resusc Emerg Med. 2016;24(8):1–7. 10.1186/s13049-016-0201-z PMC4730650 26817789

[11] Mack EH, Wheeler DS, Embi PJ (2009). Clinical decision support systems in the pediatric intensive care unit*. Pediatr Crit Care Med.

[12] Bosk CL, Dixon-Woods M, Goeschel CA, Pronovost PJ (2009). Reality check for checklists. Lancet.

[13] Hurtado JJD, Jiménez X, Peñalonzo MA, Villatoro C, de Izquierdo S, Cifuentes M. Acceptance of the WHO Surgical Safety Checklist among surgical personnel in hospitals in Guatemala city. BMC Health Serv Res. 2012;12:169–9. 10.1186/1472-6963-12-169 PMC3444374 22721269

[14] Russ SJ, Sevdalis N, Moorthy K, Mayer EK, Rout S, Caris J, Mansell J, Davies R, Vincent C, Darzi A (2015). A qualitative evaluation of the barriers and facilitators toward implementation of the WHO Surgical Safety Checklist across hospitals in England. Ann Surg.

[15] Thomassen O, Brattebø G, Heltne J-K, Søfteland E, Espeland A (2010). Checklists in the operating room: Help or hurdle? A qualitative study on health workers’ experiences. BMC Health Serv Res.

[16] Krombach JW, Edwards WA, Marks JD, Radke OC (2015). Checklists and other cognitive aids for emergency and routine anesthesia care - A survey on the perception of anesthesia providers from a large academic US institution. Anesth Pain Med.

[17] Hales BM, Pronovost PJ (2006). The checklist—a tool for error management and performance improvement. J Crit Care.

[18] Ajzen I (1991). The theory of planned behavior. Organ Behav Hum Decis Process.

[19] Godin G, Bélanger-Gravel A, Eccles M, Grimshaw J (2008). Healthcare professionals’ intentions and behaviours: A systematic review of studies based on social cognitive theories. Implement Sci.

[20] Venkatesh V, Davis F (2000). A theoretical extension of the Technology Acceptance Model: Four longitudinal field studies. Manag Sci.

[21] Francis JJ, Eccles MP, Johnston M, Walker A, Grimshaw J, Foy R, Kaner EFS, Smith L, Bonetti D (2004). Constructing questionnaires based on the theory of planned behaviour.

[22] Côté L, Turgeon J (2005). Appraising qualitative research articles in medicine and medical education. Med Teach.

[23] Côté L, Laughrea P-A (2014). Preceptors’ understanding and use of role modeling to develop the CanMEDS competencies in residents. Acad Med.

[24] Goldhaber-Fiebert SN, Howard SK (2013). Implementing emergency manuals. Anesth Analg.

[25] Di Renna T, Boet S, Crooks S (2013). The Cognitive Aids with Roles Defined – CARD: A new concept for crisis management. Resuscitation.

[26] Nelson KL, Shilkofski NA, Haggerty JA, Saliski M, Hunt EA (2008). The use of cognitive AIDS during simulated pediatric cardiopulmonary arrests. Simul Healthc.

[27] Sambasivan M, Esmaeilzadeh P, Kumar N, Nezakati H (2012). Intention to adopt clinical decision support systems in a developing country: effect of physician’s perceived professional autonomy, involvement and belief: a cross-sectional study. BMC Med Inform Decis Mak.

[28] Varonen H, Kortteisto T, Kaila M, for the EBMeDS Study Group (2008). What may help or hinder the implementation of computerized decision support systems (CDSSs): a focus group study with physicians. Fam Pract.

[29] Hor C, O’Donnell JM, Murphy AW, O’Brien T, Kropmans TJ (2010). General practitioners’ attitudes and preparedness towards Clinical Decision Support in e-Prescribing (CDS-eP) adoption in the West of Ireland: a cross sectional study. BMC Med Inform Decis Mak.

[30] Marshall S (2013). The use of cognitive Aids during emergencies in anesthesia. Anesth Analg.

[31] Dandoy CE, Davies SM, Flesch L, Hayward M, Koons C, Coleman K, Jacobs J, McKenna LA, Olomajeye A, Olson C, Powers J, Shoemaker K, Jodele S, Alessandrini E, Weiss B (2014). A team-based approach to reducing cardiac monitor alarms. Pediatrics.

